# Mast cell phenotype, TNFα expression and degranulation status in non-small cell lung cancer

**DOI:** 10.1038/srep38352

**Published:** 2016-12-06

**Authors:** A. Shikotra, C. M. Ohri, R. H. Green, D. A. Waller, P. Bradding

**Affiliations:** 1 Institute for Lung Health, Department of Infection, Immunity and Inflammation, University of Leicester, Leicester, UK; 2 University Hospitals of Leicester NHS Trust, Glenfield Hospital, Leicester, UK

## Abstract

Mast cell infiltration of tumour islets represents a survival advantage in non-small cell lung cancer (NSCLC). The phenotype and activation status of these mast cells is unknown. We investigated the mast cell phenotype in terms of protease content (tryptase-only [MC_T_], tryptase + chymase [MC_TC_]) and tumour necrosis factor-alpha (TNFα) expression, and extent of degranulation, in NSCLC tumour stroma and islets. Surgically resected tumours from 24 patients with extended survival (ES; mean survival 86.5 months) were compared with 25 patients with poor survival (PS; mean survival 8.0 months) by immunohistochemistry. Both MC_T_ and MC_TC_ in tumour islets were higher in ES (20.0 and 5.6 cells/mm^2^ respectively) compared to PS patients (0.0 cells/mm^2^) (p < 0.0001). Both phenotypes expressed TNFα in the islets and stroma. In ES 44% of MC_T_ and 37% of MC_TC_ expressed TNFα in the tumour islets. MC_T_ in the ES stroma were more degranulated than in those with PS (median degranulation index = 2.24 versus 1.73 respectively) (p = 0.0022), and ES islet mast cells (2.24 compared to 1.71, p < 0.0001). Since both MC_T_ and MC_TC_ infiltrating tumour islets in ES NSCLC patients express TNFα, the cytotoxic activity of this cytokine may confer improved survival in these patients. Manipulating mast cell microlocalisation and functional responses in NSCLC may offer a novel approach to the treatment of this disease.

Lung cancer currently causes more deaths worldwide than any other malignancy and non-small cell lung cancer (NSCLC) accounts for the majority of these cases^1^. There is increasing evidence that the immune system plays a role in the regulation of cancer development^2^,^3^,^4^, and cells of the innate and adaptive immune responses have been implicated in both the progression and curtailment of tumour growth.

Mast cells are innate immune cells which arise in the bone marrow, circulate as progenitors, and differentiate following migration into tissue. They are found in all healthy tissues, where they contribute to tissue homeostasis and host defence, but are best known for their role in allergic diseases and asthma^5^. Their primary role is to respond rapidly to a tissue insult, initiating an appropriate program of tissue inflammation and repair. However, when exposed to a chronic insult, their ongoing activation may contribute to tissue damage, remodelling and fibrosis. Mast cells are an important component of immune cell infiltrates in tumours, but their role in tumour development and progression remains unclear^6^. In many situations they have been linked with tumour progression and metastasis^7^,^8^,^9^, and this is proposed to be mediated through their ability to promote angiogenesis via the release of autacoid mediators and pro-angiogenic chemokines and growth factors^10^,^11^. For example, the products of mast cells released during degranulation have been demonstrated in co-culture to enhance the migration of cervical cancer cells^12^. Increased histamine expression has also been shown to be associated with colorectal cancer and worsening tumour stage^13^, and heparin and bound cytokines/growth factors can promote neovascularisation^14^.

Taken together, these studies suggest that degranulating mast cells may be associated with tumour progression. However, Tataroglu has suggested that there is no correlation between intratumoural mast cells and angiogenesis in NSCLC^15^, and another study found no correlation between mast cells and survival in NSCLC^16^. However, the microlocalisation of mast cells within the tumour was not assessed. In contrast, we demonstrated that while mast cell numbers are similar in the tumour stroma of patients with surgically resected NSCLC irrespective of survival status, there is a marked survival advantage when mast cells are present within clusters of NSCLC tumour epithelial cells (islets)^17^.

Mast cells exhibit marked heterogeneity across species, within different organs within the same species, and even within the same organ^5^. Heterogeneity is evident with respect to ultrastructure, receptor expression, mediator content, immunological and non-immunological activation, and pharmacological responsiveness^5^. In humans, two common mast cell phenotypes are recognised based on their protease content: mast cells which contain tryptase only (MC_T_), and mast cells containing both tryptase and chymase (MC_TC_)^18^. MC_TC_ predominate in the skin and connective tissue, and are also found in significant numbers in airway submucosal tissues^18^,^19^. MC_T_ predominate in mucosal epithelia, and are also present in the lamina propria^18^,^19^. Their roles remain unclear, but their ability to release different proteases and cytokines^18^,^19^ suggests some roles which are mutually exclusive. Mast cell phenotype has been investigated in NSCLC before by Ibaraki^20^, who concluded that MC_TC_ are associated with microvessel count, and thus, angiogenesis.

Tumour Necrosis Factor-alpha (TNFα) is an important cytokine produced by airway mast cells^21^. TNFα plays an important role in host defence and protects against cancer development as revealed by the increased incidence of cancer in patients receiving anti-TNFα therapy^22^,^23^. However, TNFα has been described by Szlosarek as having a paradoxical role in cancer, by inducing cell-mediated killing of certain tumours, as well as acting as a tumour promoter^24^. We have shown previously that increased expression of TNFα in the tumour islets of patients with NSCLC is independently associated with improved survival^25^. Tumour islet TNFα expression in extended survival patients was localised predominantly to macrophages of the M1 phenotype, and also mast cells identified by tryptase staining^25^,^26^. Whether the MC_TC_ mast cell phenotype infiltrates the tumour islets, expresses TNFα, and confers a survival advantage has not been reported.

The microanatomical localisation of mast cells within NSCLC tissue appears critical to their role in disease progression. The primary aims of the present study were therefore to define the phenotype of mast cells within NSCLC stroma and islets in terms of their protease and TNFα content and their state of activation defined by the extent of degranulation.

## Materials and Methods

### Study Population

The study was approved by the Leicestershire Research Ethics Committee (approval reference number 6529). The methods in our study were carried out in accordance with our internal standardised protocols which are relevant guidelines to our study. The tissue specimens evaluated were from 49 patients with NSCLC who had undergone resection with curative intent at the University Hospitals of Leicester National Health Service Trust (Leicester, UK). These patients had resections during two periods: one dating from 1991–1994 and the second from January to December 1999. This cohort of patients has been described previously^17^. The patients from the 1991–1994 cohort had all died at the time of the study. 4 patients from the 1999 cohort were still alive and had provided written informed consent for the use of their tissue in research at the time of surgery.

Patients were selected for the study based on their survival, without knowledge of their previous tumour mast cell counts. 24 patients had extended survival (ES) (mean ± SEM 86.5 ± 8.8 months), and 25 patients had poor survival (PS) (8.0 ± 0.78 months). Patient characteristics are summarised in Table 1.

### Immunohistology

The specimens studied were formalin-fixed and paraffin embedded. Only the advancing edge of the tumour was evaluated. Tissue sections of 4 *μ*m thickness were cut onto glass slides and then de-waxed in xylene and rehydrated through graded alcohols. Antigen retrieval was carried out using Trilogy Antigen Retrieval solution (Cell Marque, Hot Springs, USA) in a pressure cooker (heated to 117.5 °C for 1 min and then cooled to 100 °C for 30 seconds). Antibodies for phenotypic analysis were all mouse antihuman mAb as follows: tryptase (clone AA1; Dakocytomation, Ely, Cambridgeshire, United Kingdom) as a specific marker for all mast cells, chymase (clone CCL1; Abcam, Cambridge, United Kingdom) as a specific marker for mast cells expressing chymase, and TNFα (clone P/T2; Abcam, Cambridge, United Kingdom). Immunostaining was performed using the Envision double-stain kit (Dakocytomation) according to the manufacturer’s instructions and as described previously^17^. Three slides were prepared for each patient: chymase versus tryptase, tryptase versus TNFα, and chymase versus TNFα. Peroxidase and 3,3′-diaminobenzidine tetrahydrochloride (brown reaction product), and alkaline phosphatase and fast red (red reaction product) were used to label cells expressing tryptase, chymase, and TNFα. Sections were then counterstained with haematoxylin and mounted in an aqueous mounting medium (BDH Chemicals Ltd, Poole, United Kingdom). Appropriate isotype controls were performed where the primary antibodies were replaced by irrelevant mouse mAb of the same isotype and at the same concentration as the specific primary mAb.

### Analysis and Validation of Immunostaining

Analysis was performed blind with respect to the clinical outcome. The ten most representative high-power fields (x400) per slide were manually selected using an Olympus BX50 microscope (Olympus, Southall, United Kingdom). The respective areas of stroma and of tumour cell islets were then measured at x400 magnification using Scion image analysis software (Based on National Institutes of Health Image for Macintosh, modified for Windows [Scion Corp, Frederick, MD]). The number of nucleated cells with positive staining were then counted manually and expressed as cells/mm^2^ of stroma or tumour islets. Analysis was repeated for 10 patients to assess repeatability and validity. To identify mast cell phenotype, cells positive for chymase were counted as MC_TC_ and all other mast cells were counted as MC_T_. To assess the number of MC_T_ cells expressing TNFα, the number of MC_TC_ cells expressing TNFα was subtracted from the total number of tryptase + cells (i.e. the total of MC_T_ and MC_TC_) expressing TNFα.

A degranulation index score was established in order to assess the degree of degranulation by each individual mast cell as follows:

0        No degranulation

1        <33% degranulation

2        33–66% degranulation

3        >66% degranulation

### Statistical Analysis

Statistical analyses were carried out using the GraphPad Prism software package (v. 6.02; GraphPad Prism Software Inc, San Diego, CA). For categoric analysis, the median value was used as a cut point to dichotomise the series. The χ^2^ test was used to test for relationships between categoric variables, and the Mann-Whitney nonparametric test was used to compare categoric with continuous variables. Spearman’s test was used to test correlation. Kaplan-Meier survival curves were used to look for correlations with survival and were compared with the use of the log-rank statistic. For the above comparisons, p < 0.05 was considered statistically significant.

## Results

### Patient Characteristics

Of the 49 patients studied, 45 had died at the time of analysis. 31 tumours were squamous, 7 adenocarcinoma, 6 large cell, and 5 other. 27 were stage I, 17 stage II, and 5 stage IIIa. 1 patient received additional chemotherapy and 4 received additional radiotherapy for later palliation (post surgery). The patient characteristics are summarised in Table 1.

### Validation of Analysis

**Figure 1:**
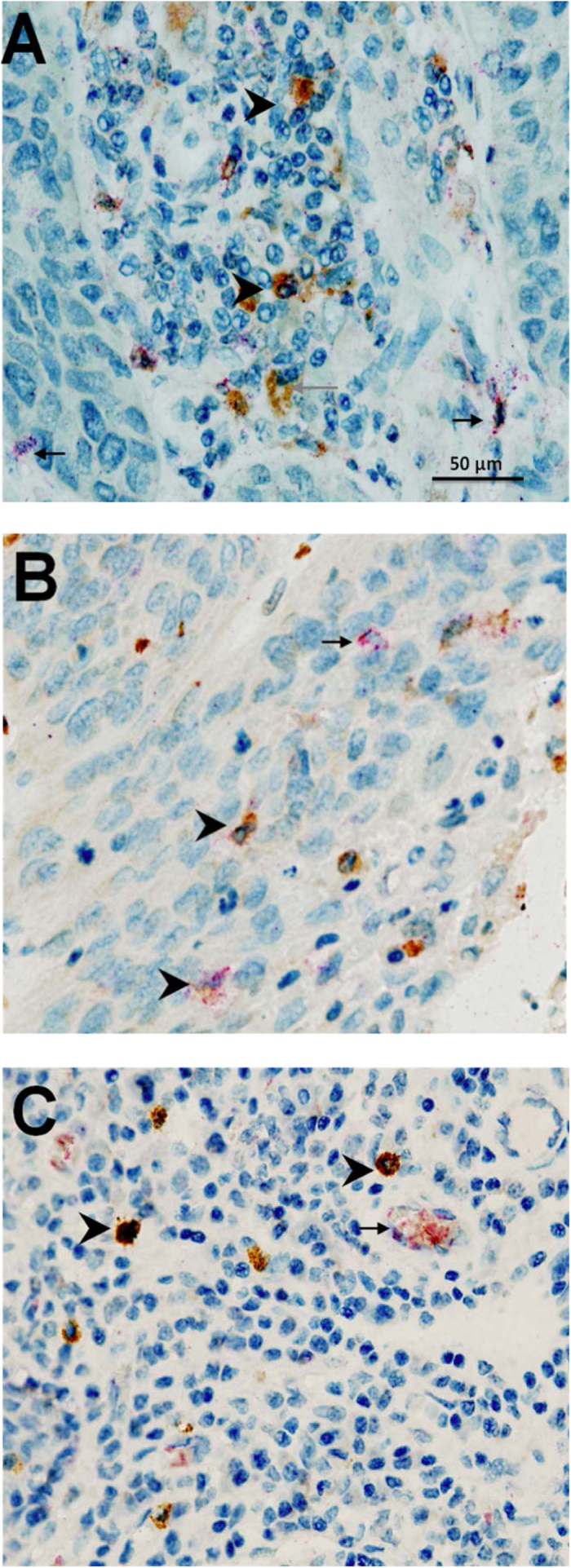
Examples of immunohistochemical double-staining for (**A**) chymase (brown) and tryptase (red) demonstrating the presence of MC_T_ mast cells (red) and MC_TC_ (reddish brown) (**B**) tryptase (brown) and TNFα (red) demonstrating the presence of TNFα in tryptase + mast cells, and (**C**) chymase (brown) and TNFα (red) demonstrating the expression of TNFα in MC_TC_ mast cells. Arrowhead = double-stain cell. Black arrow = single-stain red cell. Grey arrow = single-stain brown cell.

Clear and distinguishable staining was evident for tryptase, chymase and TNFα, and double-stained cells were readily identifiable (Fig. 1). Appropriate isotype controls were negative. Cells counts were repeated and the intraclass correlation coefficient was calculated as 0.797 (p < 0.01). This method of analysis has also been validated by our group previously^17^. The degranulation index was validated by two separate observers with an intraclass correlation of 0.668 (p < 0.05).

### Cellular Distribution

**Figure 2:**
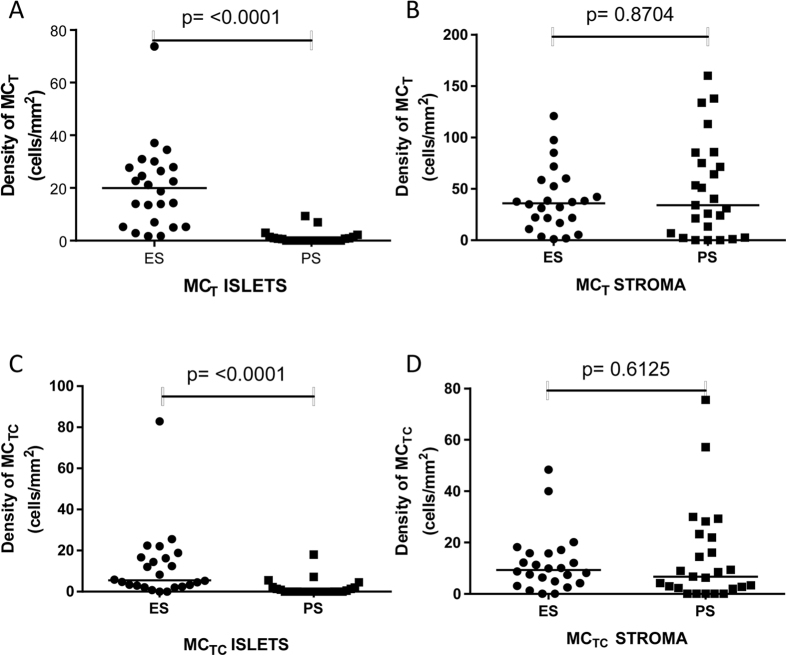
Mast cell densities for MC_T_ in the islets (**A**) and stroma (**B**) and for MC_TC_ in the islets (**C**) and stroma (**D**) in extended survival (ES) and poor survival (PS) patients.

There were significantly more MC_T_ in the tumour islets of the ES group (median, 20.0 cells/mm^2^ [IQR 5.7–27.9] compared to the PS group (median, 0.0 [IQR 0–1.2], p < 0.0001) (Fig. 2A). In contrast, in the stroma there was no significant difference between the MC_T_ densities in the ES group (median, 36.0 cells/mm^2^ [IQR 17.9–57.06]) and the PS group (median, 34.1 cells/mm^2^ [IQR 4.6–80.1], p = 0.87) (Fig. 2B).

There were also significantly more MC_TC_ in the tumour islets of the ES group (median, 5.6 cells/mm^2^ [IQR 2.6–16.6]) compared to the PS group (median, 0 [IQR 0–1.7], p = < 0.0001) (Fig. 2C). In contrast, in the stroma there was no significant difference between the MC_TC_ densities in the ES group (median, 9.3 cells/mm^2^ [IQR 4.4–15.8]) and the PS group (median, 6.7 cells/mm^2^ [IQR 2.1–22.6], p = 0.61) (Fig. 2D). The percentage of mast cells which were MC_T_ or MC_TC_ in the tumour compartments are shown in Table 2.

**Figure 3:**
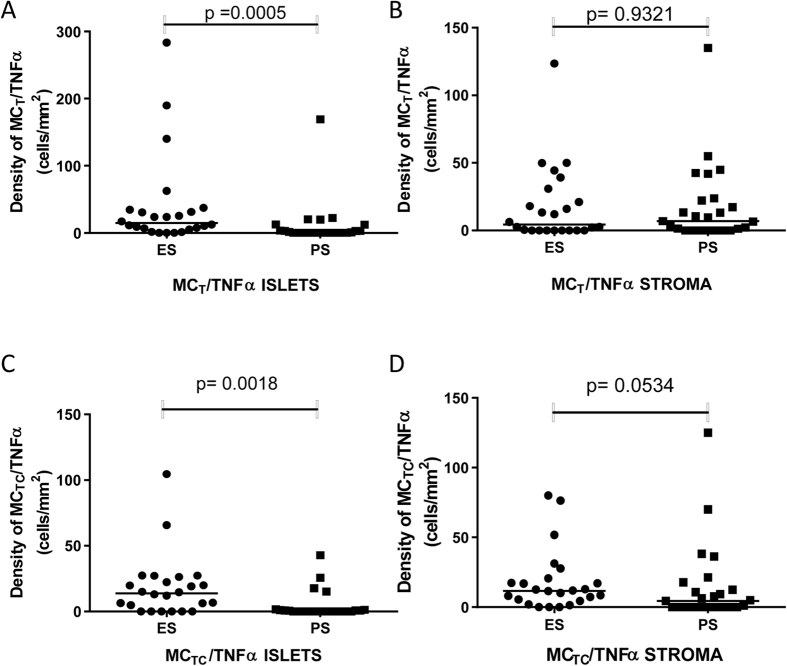
Double-stain densities of MC_T_/TNFα in the islets (**A**) stroma (**B**) and MC_TC_/TNFα in the islets (**C**) and stroma (**D**) in extended survival (ES) and poor survival (PS) patients. MC_T_/TNFα densities were calculated by subtracting the chymase (MC_TC_) count from the tryptase (total mast cells) count.

The median density of MC_T_ and MC_TC_ expressing TNFα in the tumour islets of patients with ES was significantly greater (median 14.9 [IQR 5.6–33.76] and median 13.8 cells/mm^2^ [IQR 1.2–25.3] respectively) than in patients with PS (median 0.1 [IQR 0–7.9] and 0.0 cells/mm^2^ [IQR 0–1.2] respectively) (p = 0.0005 for MC_T_ & p = 0.0018 for MC_TC_) (Fig. 3A and C). The median density of MC_T_ and MC_TC_ expressing TNFα in the stroma of patients with ES was 4.5 (IQR 0–28.5) and 11.7 (IQR 4.8–19.9) cells/mm^2^ respectively compared to 6.9 (range 0–23.1) and 4.4 (IQR 0–15.2) cells/mm^2^ respectively in with patients with PS (p = 0.932 for MC_T_ and p = 0.053 for MC_TC_) (Fig. 3B and D). The median density of all cells expressing TNFα (mast cells and other cells) in tumour islets of patients with an above median survival was also noted to be significantly greater (71.1 cells/mm^2^) than in patients with a below median survival (12.7 cells/mm^2^) (p = 0.0035). The proportion of MC_T_ and MC_TC_ which expressed TNFα in the different tissue compartments are shown in Table 2.

### Degranulation Index

**Figure 4:**
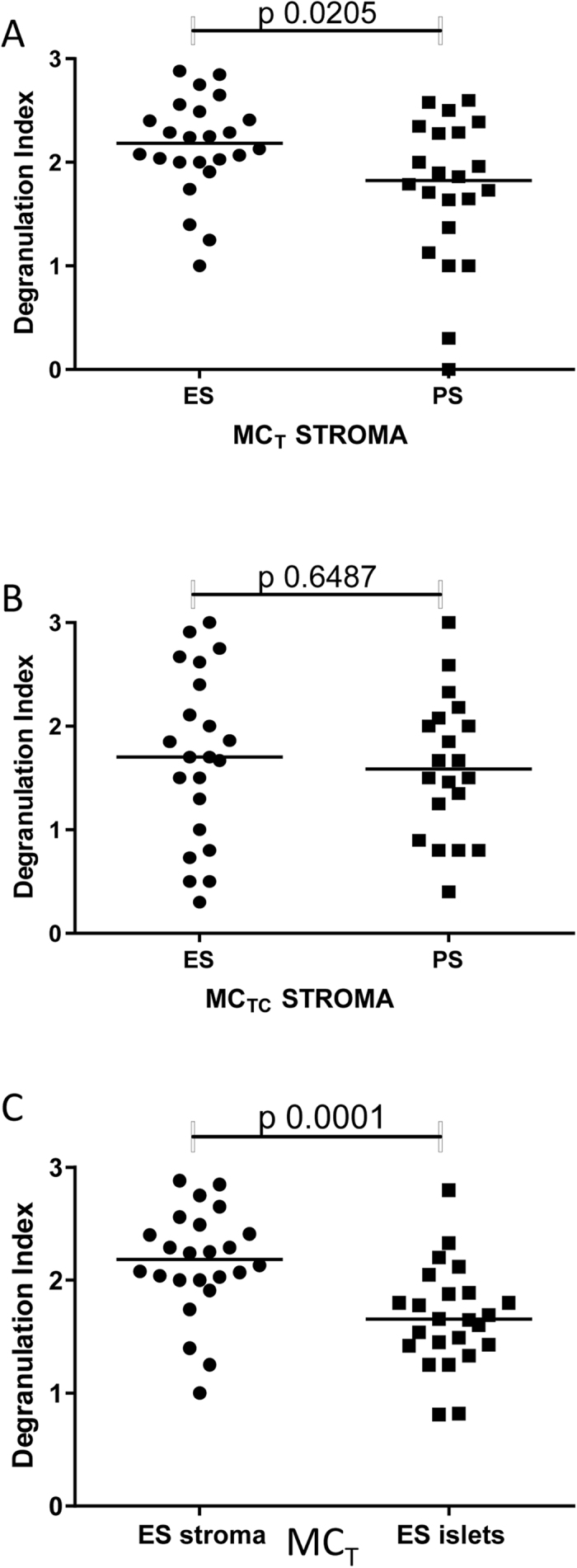
Degranulation Index of MC_T_ in the tumour stroma (**A**) MC_T_ in tumour stroma versus islets (**B**) and MC_TC_ in the tumour stroma (**C**).

In the stroma, MC_T_ were degranulated to a greater degree in patients with ES than in those with PS (Fig. 4A, median [IQR] degranulation index 2.2 [2.0–2.5] compared to 1.83 [1.3–2.3], p = 0.021). There was no significant difference between ES and PS mast cell degranulation index for MC_TC_ in stroma (Fig. 4B). MC_T_ in the ES stroma were also more degranulated than MC_T_ in the ES islets (degranulation index median 2.2 [IQR 2.0–2.5] compared to 1.7 [IQR 1.4–1.9], p < 0.0001) (Fig. 4C). There were insufficient cells for analysis of degranulation in the islets of PS patients.

### Kaplan-Meier Survival Analysis

**Figure 5:**
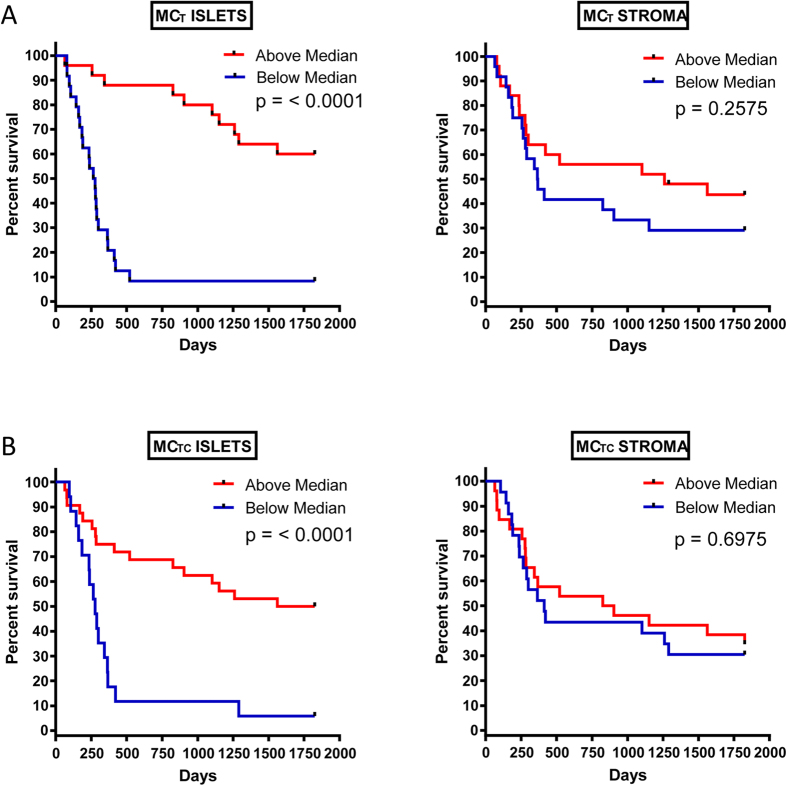
Kaplan-Meier five-year survival curves for MC_T_ in the islets and stroma (**A**) and MC_TC_ in the islets and stroma (**B**). Subjects were divided at the median value.

**Figure 6:**
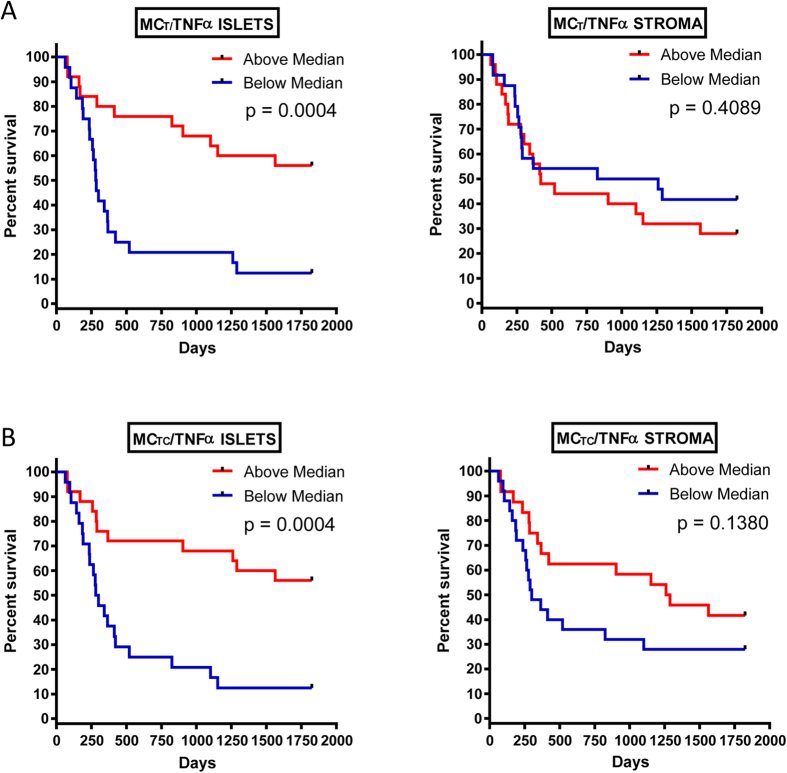
Kaplan-Meier five-year survival curves for MC_T_/TNFα in the islets and stroma (**A**) and MC_TC_/TNFα in the islets and stroma (**B**).

For further analysis, the data were divided into two groups above and below the median cell count values. Kaplan-Meier survival curves were plotted to investigate further the association of cell densities with survival. The log rank statistic was used to compare survival rates. In the tumour islets, patients with above median density of both MC_T_ (Fig. 5A) and MC_TC_ (Fig. 5B) had significantly greater predicted survival (p < 0.0001) but there was no correlation with stromal mast cell densities and survival (Fig. 5A and B). There was a positive association between survival and tumour islet density of mast cells (MC_T_ and MC_TC_) expressing TNFα (p = 0.0004) (Fig. 6A and B). In contrast there were no survival differences evident using the same analysis on stromal cell counts (Fig. 6A and B).

## Discussion

We have shown previously that mast cell infiltration of tumour islets in surgically resected NSCLC confers a marked survival advantage independently of tumour stage^17^. This study extends those findings by delineating the phenotype of mast cells within the tumour stroma and epithelial islets, and the extent of their degranulation. Of potential importance, both protease phenotypes are present within the tumour islets of patients with ES, they express TNFα, and the presence of both is associated with increased survival following surgical resection.

In healthy human airways mast cells are located predominantly in the lamina propria, and the density of mast cells there is similar to the density recorded here in the NSCLC tumour stroma^21^. Furthermore, the normal airway lamina propria ratio of MC_T_:MC_TC_ is approximately 80:20^19^,^27^, which is again similar to that in the NSCLC stroma. This suggests that the homeostatic mechanisms regulating mast cell density and phenotype is similar in healthy bronchus and NSCLC stroma. Mast cells are rarely found in healthy airway epithelium, but those present have been reported to be almost exclusively of the MC_T_ phenotype^18^. In steroid-naïve asthma, mast cells infiltrate the airway epithelium with an MC_T_:MC_TC_ ratio of about 80:20^19^. This presumably occurs under the influence of epithelial-derived chemoattractants which are released in response to inhaled pro-inflammatory stimuli. It is interesting therefore that in NSCLC epithelial islets, mast cells are also present in some patients, that their presence correlates with ES, and that their protease distribution of 80:20 MC_T_:MC_TC_ is similar to that seen in asthmatic airway epithelium. This suggests that in patients with ES, the tumour epithelium expresses a repertoire of chemoattractants, growth factors and cell-cell signals for the recruitment, differentiation and survival of both the MC_T_ and MC_TC_ phenotype, and that common mechanisms may exist for the recruitment of these cells in both asthma and NSCLC.

The correlation of improved survival with both MC_T_ and MC_TC_ mast cell phenotypes within the NSCLC tumour islets suggests that mast cells contribute to the anti-tumour immunological response, which also comprises infiltration by CD68+ macrophages^17^, natural killer and regulatory T cells^28^. There are many mast cell products which may contribute to the recruitment and activation of these other immune cells, but TNFα may be particularly important. This cytokine is stored pre-formed and secreted rapidly by mast cells in response to numerous diverse stimuli including IgE-dependent activation^29^,^30^, TLR-dependent activation^31^ and cell-cell contact with T cells^32^. It is therefore likely that the expression of TNFα by mast cells of both the MC_T_ and MC_TC_ phenotypes within the tumour islets has important biological consequences.

We have shown previously that the expression of TNFα in the tumour islets is also associated with improved survival^25^. These findings suggest that TNFα has a protective role when present in the NSCLC tumour islets, contributing to the limitation of tumour growth and dissemination. In mouse models mast cell-derived TNFα significantly increased T cell proliferation and cytokine production^33^ and IgE- and antigen-dependent mast cell enhancement of T cell activation required TNFα^34^. TNFα also induces cytotoxic activity in macrophages, and so mast cell-derived TNFα may play a pivotal role in the cytokine pathways influencing cytotoxic T cells and macrophages within the tumour islets, and thus have important consequences on cytotoxicity against tumour cells. This would be in keeping with other recently identified protective roles for mast cells in mammalian systems^35^.

We found no correlation between the density of MC_TC_ and poor survival which conflicts with the results of Ibaraki and colleagues^20^ who reported a relationship between MC_TC_ density and microvessel density in NSCLC. This difference may be due to the fact that in that study no assessment of the microlocalisation of mast cells within the tumour was made. In addition, we noted that patients with poor survival had low numbers of both phenotypes of mast cell within their tumour islets, in keeping with our previous findings^17^.

Our results also demonstrate that patients who have a better outcome have greater degranulation of their MC_T_ subset in the NSCLC stroma. It is plausible that granular products such as heparin and proteases are able to disrupt the stroma after degranulation, thus inhibiting consequent tumour growth. Mast cell proteases are known to cause cell structural alterations and loss of the extracellular matrix integrity^6^. It is attractive to hypothesise that degranulation is the default and potentially protective mast cell anti-tumour response, but in poor prognosis patients there is inhibition of this with concomitant enhancement of detrimental factors such as pro-angiogenic cytokine production. In support of this, cyclooxygenase-2 and its product PGE_2_ are over-expressed in NSCLC^36^,^37^, and correlate with poor survival^38^. PGE_2_ is known to inhibit human lung mast cell degranulation^39^, but increase the synthesis and release of VEGF^40^,^41^. In addition, PGE_2_ is an inhibitor of human lung mast cell migration^42^, and so the increased production of PGE_2_ by poor prognosis tumours might also explain why these tumours fail to recruit mast cells to the tumour islets. Thus the expression of PGE_2_ in NSCLC and other tumours could be a key factor determining whether mast cells are protective or pro-tumorigenic, and explain the discrepancies in previous studies with regards to their role.

It is recognised that mast cells exhibit heterogeneity with regards to their cytokine content. In the lung, the MC_T_ phenotype expresses predominantly IL-5 and IL-6, while the MC_TC_ phenotype expresses IL-4 and IL-13^19^. This study demonstrates that in lung cancer at least, mast cells of both the MC_T_ and MC_TC_ phenotype express TNFα. The factors determining the expression of these cytokines in the MC_T_ versus MC_TC_ phenotype are not known and require further study.

In summary, we have shown that the density of the two mast cell phenotypes MC_T_ and MC_TC_ is increased in the tumour islets of patients with ES in NSCLC. The production of TNFα by both mast cell phenotypes within the tumour islets, and the degranulation of the MC_T_ phenotype within the tumour stroma, may be particularly important for their interaction with other immune cells and the associated inhibition of tumour progression.

Manipulating mast cell microlocalisation and functional responses in NSCLC, for example through the inhibition of PGE_2_ production using cyclooxygenase-2 inhibitors, may offer a novel approach to the treatment of this disease.

## Additional Information

**How to cite this article**: Shikotra, A. *et al*. Mast cell phenotype, TNFα expression and degranulation status in non-small cell lung cancer. *Sci. Rep.* **6**, 38352; doi: 10.1038/srep38352 (2016).

**Publisher's note:** Springer Nature remains neutral with regard to jurisdictional claims in published maps and institutional affiliations.

## Figures and Tables

**Table 1:** Patient demographics.

Characteristic		Extended Survival	Poor Survival	p value
No. of patients		24	25	
Age – years¥		65.6 ± 1.9	69.7 ± 1.4	0.0827
Male sex – no. (%)#		17 (70.8)	19 (76)	0.7536
Year of surgery – no. (%)#				
	1991	0 (0)	1 (4)	0.8106
	1992	2 (8)	1 (4)	
	1993	3 (13)	3 (12)	
	1994	6 (25)	4 (16)	
	1999	13 (54)	16 (64)	
Tumour stage – no. (%)#				
	1	12 (50)	15(60)	0.4203
	2	8 (33)	9 (36)	
	3a	4 (17)	1 (4)	
Histology – no. (%)#				
	Squamous	17 (71)	14 (56)	0.7709
	Adenocarcinoma	3 (13)	4 (16)	
	Large cell	2 (8)	4 (16)	
	Other	2 (8)	3 (12)	
Tumour Grade – no. (%)#				
	Well	1 (4)	3 (12)	0.0510
	Moderate	12 (50)	5 (20)	
	Poor	10 (42)	17 (68)	
	Not recorded	1 (4)	0 (0)	
Adjuvant Chemotherapy (%)		1 (5)	0 (0)	
Radiotherapy (%)		2 (10)	2 (10)	
Palliative Radiotherapy (%)		2 (10)	2 (10)	
Survival – months¥		86.5 ± 8.8	8.0 ± 0.78	**<0.0001**

*Plus-minus values are means* + *SEM.*

^¥^Unpaired T test.

^#^Fischer’s Exact Test.

**Table 2:** The percentage of total mast cells positive for each phenotype (MC_TC_ or MC_T_), and the percentage of each phenotype expressing TNFα in the islets of extended survival patients (ES) and poor survival patients (PS) and stroma of extended survival patients (ES) and poor survival patients (PS).

	ES Islets	PS Islets	ES Stroma	PS Stroma
MC_TC_	**27.06**(0–93.7)	#	**23.5**(0–91.7)	**17.6**(0–100)
MC_T_	**72.94**(3.4–100)	#	**76.5**(8.3–100)	**82.4**(0–100)
MC_TC_/TNFα	**36.8**(0–100)	#	**52.6**(0–100)	**20.0**(0–100)
MC_T_/TNFα	**43.6**(0–100)	#	**40.5**(0–100)	**87.5**(0–100)

Median values are shown with (range).

^#^Insufficient cells for analysis.
